# The Role of Minoxidil in Treatment of Alopecia Areata: A Systematic Review and Meta-Analysis

**DOI:** 10.3390/jcm13247712

**Published:** 2024-12-17

**Authors:** Michał Majewski, Karolina Gardaś, Anna Waśkiel-Burnat, Michał Ordak, Lidia Rudnicka

**Affiliations:** 1 Department of Dermatology, Medical University of Warsaw, Nowogrodzka 59 Str., 02-014 Warsaw, Poland; s082570@student.wum.edu.pl (M.M.); s082430@student.wum.edu.pl (K.G.); lidia.rudnicka@wum.edu.pl (L.R.); 2 Department of Pharmacotherapy and Pharmaceutical Care, Faculty of Pharmacy, Medical University of Warsaw, Banacha 1 Str., 02-097 Warsaw, Poland; michal.ordak@wum.edu.pl

**Keywords:** alopecia, hair loss, alopecia areata, minoxidil, oral minoxidil, topical minoxidil, treatment, systematic review, meta-analysis

## Abstract

**Background/Objectives**: Minoxidil, in addition to its vasodilatory effect, has also immunomodulatory properties that may be partially responsible for its efficacy in alopecia areata. The aim of the study was to evaluate the efficacy of monotherapy with topical or oral minoxidil in alopecia areata. **Methods**: A systematic review and meta-analysis of the efficacy of monotherapy with minoxidil in alopecia areata was conducted following the PRISMA guidelines. Efficacy of minoxidil in alopecia areata was evaluated separately for three groups of the patients: (1) treated with 5% topical minoxidil, (2) less than 5% topical minoxidil, and (3) oral minoxidil. Therapeutic response was defined as any regrowth of terminal hair. **Results**: Of 244 articles, 13 were considered eligible for the further analysis. The study included 372 patients with alopecia areata (338 using topical minoxidil and 34 taking oral minoxidil). The mean time of treatment ranged from 2 to 60 weeks (mean: 27 weeks). The response rate for 5% topical was 82% (95% CI 0.7–0.93) and 58% (95% Cl 0.5–0.67) for the less than 5% topical minoxidil group. For the group of patients treated orally, the response rate was 82%. **Conclusions**: Minoxidil, both topical and oral, may be beneficial in monotherapy in patients with alopecia areata. 5% topical minoxidil is characterized by significantly higher efficacy compared to minoxidil at a lower concentration. There are no sufficient data to recommend minoxidil as a first-line therapeutic option for alopecia areata.

## 1. Introduction

Alopecia areata is an autoimmune form of non-scarring hair loss that may affect any hair-bearing area [1]. The disease is diagnosed in approximately 2% of the overall population, irrespective of ethnicity, gender, or age group [2]. The presentation of alopecia areata is variable. The disease commonly begins as patchy hair loss, which can progress to more extensive forms with complete scalp (alopecia totalis) or scalp and body hair loss (alopecia universalis) [3].

The exact pathogenesis of alopecia areata is only partially explained. The role of genetic and immunological factors, allergy, and microbiome is discussed [4,5]. In the initial phases of alopecia areata, the overproduction of interferon-gamma (IFN-γ) induces the expression of major histocompatibility complex (MHC) class I in hair follicles by upregulating chemokines of the C-X-C motif (CXCL) and intracellular adhesion molecules (ICAM). It leads to the immune recognition of hair follicle autoantigens by autoreactive cytotoxic CD8 + NKG2D + T cells, resulting in hair loss [6,7,8,9].

Various therapeutic agents are used in alopecia areata, such as topical, intralesional, and systemic glucocorticosteroids; topical immunotherapy; cyclosporine; methotrexate; and Janus kinase inhibitors. Both topical and oral minoxidil are considered adjuvant therapy [10].

Minoxidil was introduced in 1968 to treat hypertension [11]. Hypertrichosis was observed in approximately 20% of patients who received minoxidil, which prompted further research on the drug in the treatment of alopecia. To date, 2% and 5% minoxidil solutions are approved by the Food and Drug Administration (FDA) for the treatment of androgenetic alopecia. Nevertheless, minoxidil, both oral and topical, is commonly used in other forms of hair loss.

Minoxidil as a therapeutic option for alopecia areata was first described in 1982 [12]. Since then, numerous studies considering the role of minoxidil in alopecia areata have been published.

There is a lack of studies evaluating the effectiveness of minoxidil in monotherapy for alopecia areata. Many studies assess the effect of only topical minoxidil at a single concentration (most commonly 5%) or as a part of combination therapy, making it difficult to perform a comprehensive analysis.

## 2. Objectives

The aim of the study was to assess the efficacy of monotherapy with topical or oral minoxidil in patients with alopecia areata.

## 3. Materials and Methods

A systematic review and meta-analysis of the literature on the use of minoxidil as monotherapy in patients with alopecia areata was performed by searching the PubMed database, with the last search on 17 August 2023. The terms used for the search were “alopecia areata”, “alopecia totalis”, or “alopecia universalis” combined with “minoxidil”. In addition, the reference sections of all relevant articles were searched for further publications. The articles were screened by two independent researchers (M.M. and K.G). The systematic review was performed according to the Preferred Reporting Items for Systematic Reviews and Meta-Analyses (PRISMA) guidelines [13]. Inclusion criteria were the use of minoxidil in monotherapy in patients with alopecia areata. Written in English full-text papers of case clinical trials meeting eligibility criteria were included. The exclusion criteria were incomplete data, such as unknown number of patients or therapeutic outcomes and animal studies. Articles that did not report response rates or which did not allow for response rates to be calculated were also eliminated from the analysis. Case reports, letters to the editor, reviews, opinions, commentaries, or editorials were rejected. Abstracts as post-conference material were excluded. Studies in which patients received other therapies such as corticosteroids or cyclosporine were also excluded. If data were duplicated in more than one study, the most complete study was included in the analysis.

Data on the number of patients, sex, age, form of alopecia areata (patchy alopecia areata, alopecia totalis, or universalis), and duration of treatment were extracted and collected. Efficacy of minoxidil in alopecia areata was evaluated separately for three groups of the patients: (1) treated with 5% topical minoxidil, (2) treated with less than 5% topical minoxidil, and (3) treated with oral minoxidil. Therapeutic response was defined as any regrowth of terminal hair. Regrowth with the presence of only intermediate and/or vellus hair was considered a lack of response to treatment. Additionally, in studies where the authors provide information on percentage hair regrowth, the results were classified into three groups: (1) hair regrowth equal to or less than 30%, (2) hair regrowth between 30% and 70%, and (3) hair regrowth equal to or greater than 70%.

The meta-analysis was conducted using the statistical software PQStat, version 1.8.0. Heterogeneity across studies was assessed using three statistical measures: the Q statistic, the H^2^ coefficient, and the I^2^ statistic. The Q statistic was calculated, and its *p*-value determined whether heterogeneity between studies was statistically significant. The H^2^ coefficient and its confidence interval were used to evaluate the degree of heterogeneity. If the confidence interval included 1, it was assumed that there was no significant variability between studies. The I^2^ statistic was calculated to quantify the proportion of total variability attributable to heterogeneity, with values near 50% interpreted as moderate heterogeneity. The outcome was a summary proportion. Publication bias was examined using the Egger regression test. In addition, sensitivity analyses were performed by excluding individual studies one at a time to assess their influence on the summary proportion and heterogeneity measures. These analyses allowed identification of studies with a disproportionate impact on the overall results.

## 4. Results

**Figure 1:**
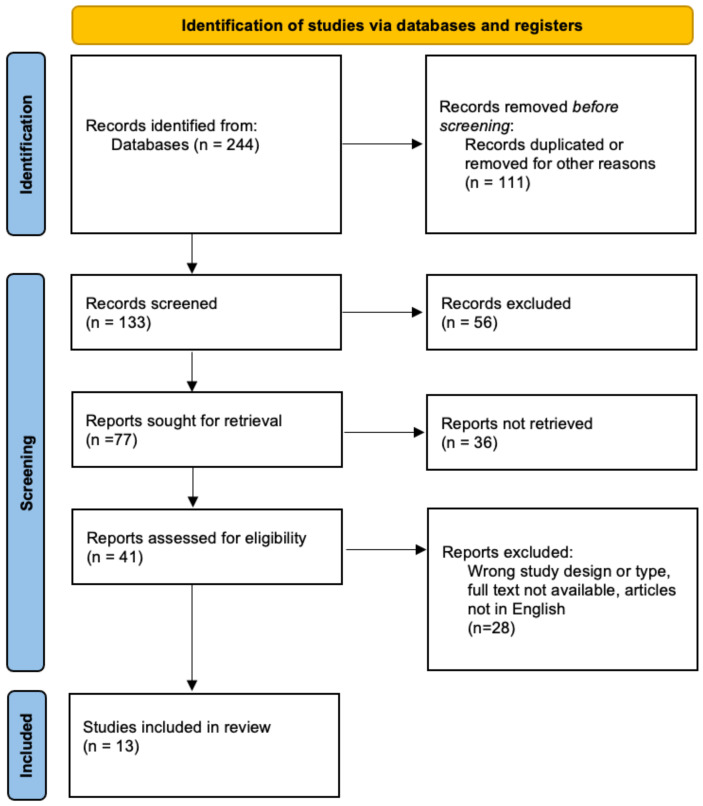
Flow chart diagram illustrating the database searches, number of publications identified, screened, and final full texts included in the systematic review.

Of an initial selection of 244 articles, 13 publications were finally selected for the final analysis: 12 concerning topical minoxidil [14,15,16,17,18,19,20,21,22,23,24,25] and one oral minoxidil [26]. The data flow diagram is presented in Figure 1.

In total, 372 patients with alopecia areata were included in the analysis: 338 patients (117 women and 106 men) treated with topical and 34 patients (19 women and 15 men) with oral minoxidil. The age of the patients ranged from 3 to 81 years (mean: 31 years). The treatment duration ranged from 2 to 60 weeks (mean: 27 weeks).

In the topical minoxidil group, 174 (38 women and 39 men) patients were treated with 5% topical minoxidil [14,15,16,17,19,20,21], 65 patients (32 women and 29 men) with 3% topical minoxidil [18,21,23], and 99 patients (28 women and 23 men) with 1% topical minoxidil [22,24,25]. The duration of the treatment ranged from 2 to 60 weeks (mean: 29 weeks).

There was only one study on the use of oral minoxidil in patients with alopecia areata. In the study performed by Fiedler-Weiss et al. [26], oral minoxidil was used in 34 patients (19 women and 15 men) with alopecia areata at a relatively high daily dose of 10 mg. The treatment duration was 24 weeks.

Studies included in the review are presented in Table 1.

### 4.1. Meta-Analysis

Only patients from the 5% topical and less than 5% topical minoxidil groups were used for meta-analysis. Patients taking oral minoxidil were not included due to their insufficient population size.

In total, 12 studies were used for meta-analysis with the objective of comparing the efficacy between 5% concentration minoxidil and a concentration lower than 5%.

#### 4.1.1. 5% Topical Minoxidil

**Figure 2:**
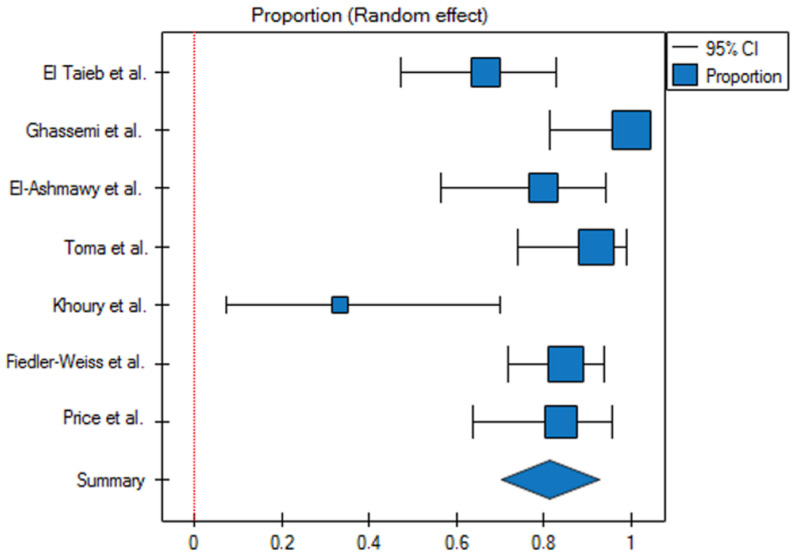
Analysis of proportions for therapeutic response in patients treated with 5% topical minoxidil (*p* < 0.01). Cl—confidence interval.

**Figure 3:**
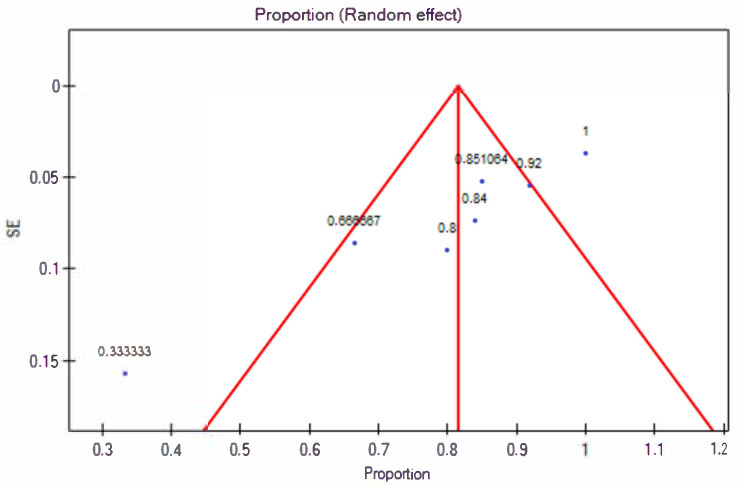
Assessment of risk of bias for 5% topical minoxidil treatment (Egger test, Egger’s coefficient (b) = 5.18 and *p* = 0.001; the presence of publication bias). SE—standard error.

The response rate for the 5% topical minoxidil group was 82% (95% CI 0.7–0.93) (Figure 2). The prediction interval for the variable effect ranged from 0.45 to 1. The H2 coefficient is 5.1 (95% CI 2.51–10.36). The Q statistic is 30.59 with a *p*-value of 0.00003, which indicates substantial heterogeneity among the studies. The I2 is 80.39% (95% CI 60.16–90.35%). The studies are asymmetric, with an Egger’s coefficient (b) of −5.18 and a *p*-value of 0.001. Based on the analysis of the funnel plot, it can be concluded that two studies responsible for publication bias are Ghassemi et al. [15] and Khoury et al. [19] (Figure 3).

#### 4.1.2. Less than 5% Topical Minoxidil

**Figure 4:**
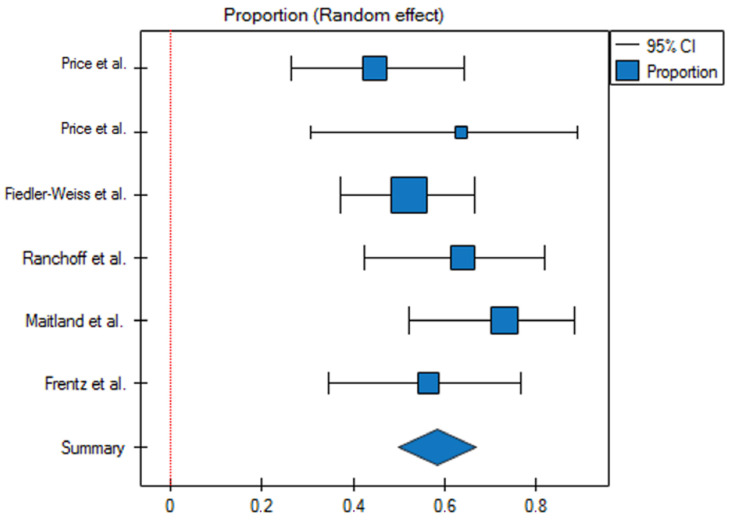
Analysis of proportions for therapeutic response in patients treated with less than 5% topical minoxidil (*p* = 0.28). Cl—confidence interval.

**Figure 5:**
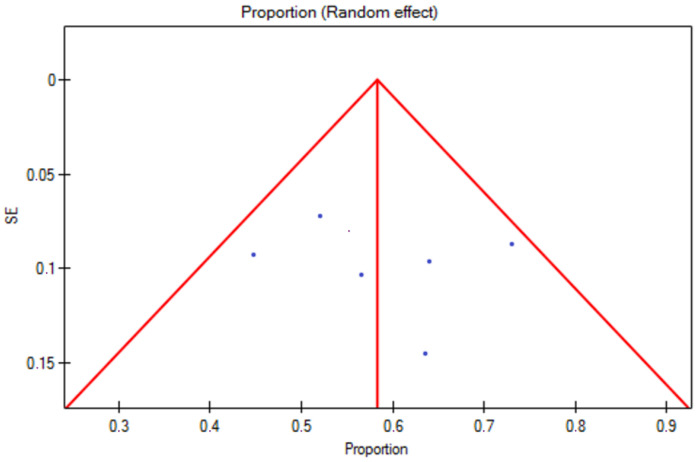
Assessment of risk of bias for less than 5% topical minoxidil treatment (Egger test, Egger’s coefficient (b) =1.21 and *p* = 0.66; no evidence of bias). SE—standard error.

In total, 164 patients treated with less than 5% minoxidil were included in the meta-analysis. For the group of patients using minoxidil at a concentration lower than 5%, the response rate was 58% (95% Cl 0.5–0.67), and the prediction interval for the variable effect was 0.4–0.76 (Figure 4). After including all studies, the assumption of heterogeneity is met: the Q statistic is 6.27 with a *p*-value of 0.28. The H2 coefficient is 1.24 (95% CI 0.55–2.85), which indicates low heterogeneity among the studies. The I2 is 20.29% (95% CI 0–64.89%). The Egger’s coefficient (b) is 1.21, with a *p*-value of 0.66. This indicates that the studies are symmetric (no evidence of bias) (Figure 5).

### 4.2. Treatment Outcomes Based on Percentage of Hair Regrowth

In total, hair regrowth equal to or more than 70% was achieved in 19/58 (33%) patients treated with topical minoxidil. Hair regrowth equal to or more than 70% was more common in patients treated with 5% topical minoxidil (13/25, 52%) compared to patients treated with minoxidil at a lower concentration (3/11, 27% and 3/22, 14%) (Table 2).

## 5. Discussion

Minoxidil is an antihypertensive drug that stimulates hair growth and reduces hair loss. It induces the opening of ATP-dependent potassium channels in vascular smooth muscle cells, which results in their relaxation and, consequently, vasodilation [27,28,29]. It leads to an increased cutaneous blood flow, resulting in enhanced oxygen and growth factor delivery to the hair follicle [30]. Minoxidil also stimulates prostaglandin E2 (PGE2) and leukotriene B4 (LTB4) production, which may enable hair follicles to grow continuously, maintain the anagen phase, and shorten the telogen phase by activating the Wnt/beta-catenin pathway [31,32,33]. Moreover, Zachary et al. [34] and Yano et al. [35] established that minoxidil stimulates the expression of vascular endothelial growth factor (VEGF), which increases perifollicular vascularization and accelerates hair growth in a dose-dependent manner [36].

Minoxidil also has an immunomodulatory effect. It moderates concanavalin A (an intermediary in the T lymphocyte activation process), which causes suppression of T-cells [37,38,39,40,41]. Minoxidil acts as a proliferative and anti-apoptotic agent on human dermal papilla cells by triggering the activation of ERK and Akt pathways. Moreover, it prevents cell death by elevating the Bcl-2/Bax ratio [42]. Finally, minoxidil causes significant downregulation of IL-1α gene expression [43]. IL-1α is a direct growth inhibitory agent in hair follicles and plays an important role in the pathogenesis of alopecia areata [44].

The study performed by Buhl et al. [45] on organ cultures of vibrissa follicles presented that the sulfated metabolite of minoxidil has a greater pore potent stimulatory effect on the proliferation of hair follicle epithelial cells. The conversion of minoxidil to its active form, minoxidil sulfate, is mediated by follicular sulfotransferase, and variability in the response rate to minoxidil is partly due to differences in sulfotransferase levels [46].

**Figure 6:**
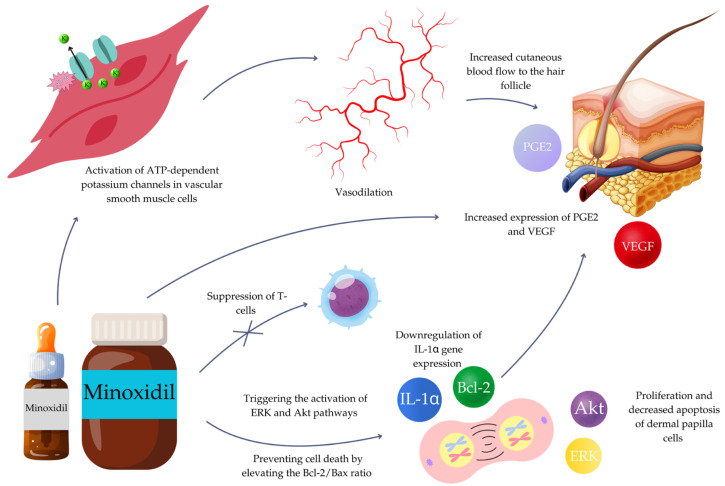
The mechanism of action of minoxidil in alopecia areata treatment. ATP—adenosine triphosphate; Bcl-2—B-cell lymphoma 2; ERK—extracellular signal-regulated kinase; IL-1α—interleukin-1 alpha; PGE2—prostaglandin E2; VEGF—vascular endothelial growth factor.

The mechanism of action of minoxidil in alopecia areata treatment has been summarized in Figure 6.

Minoxidil is usually recommended as an adjuvant therapy for alopecia areata [47]. It has been shown that the combination of tofacitinib and oral minoxidil may be more efficacious than tofacitinib in monotherapy [48]. Moreover, synergistic effects of minoxidil have been observed with laser and light therapy [49], as well as anthralin [50].

Our meta-analysis revealed that both topical and oral minoxidil monotherapy can be effective in the treatment of alopecia areata.

In alopecia areata, 5% topical minoxidil was characterized by significantly higher efficacy compared to minoxidil at a lower concentration. This observation is consistent with those observed in patients with androgenetic alopecia [51].

Minoxidil typically begins to show results within four to eight weeks, but significant improvements in hair growth may take over four months of continuous use. In alopecia areata, many medications demonstrate effectiveness that gradually improves over time [52]. The present analysis showed that the efficacy of topical minoxidil in alopecia areata may depend on the duration of treatment. In patients with alopecia areata treated with 5% topical minoxidil for more than 28 weeks, higher response rates (84–85%; mean: 85%) compared to patients treated for shorter periods (33–100%; mean: 74%) were observed.

Topical minoxidil is generally not recommended for patients with alopecia totalis or alopecia universalis, as many studies confirm their lack of efficacy in these types of alopecia [53,54]. Indeed, in the study performed by Maitland et al. [24], 53% of patients (8/15) with patchy alopecia areata treated with 5% topical minoxidil had terminal hair regrowth, whereas only in 17% (1/6) of subjects with alopecia totalis and 29% (2/7) with alopecia universalis was terminal hair regrowth observed [24].

The present analysis did not reveal higher efficacy of oral minoxidil at a daily dosage of 10 mg over 5% topical minoxidil in patients with alopecia areata (mean response rate: 82% vs. 83%, respectively). However, in the study in which patients received oral minoxidil, the patient group included individuals with severe, treatment-resistant forms of the disease who showed either no response or cosmetically inadequate response to 5% topical minoxidil [26]. It should be emphasized that minoxidil at the dose of 10 mg daily is not considered as low-dose therapy. Its use can be associated with a risk of systemic side effects (such as hypotension, arrhythmia, and peripheral edema).

A key limitation of this review is the small number of studies, particularly those investigating oral minoxidil, underscoring the necessity for future research to expand the evidence base. Other limitations are variabilities in study design and quality, lack of long-term data, possible publication bias, and the absence of randomization in some studies. The lack of large clinical trials constrains the ability to reach conclusive findings. The absence of comprehensive patient-level data limits our ability to perform detailed comparisons of the studied populations, restricting such analyses only to the data published by the authors. Additionally, minoxidil is frequently used in combination with other medications in alopecia areata, which complicates the assessment of its efficacy as a monotherapy.

## 6. Conclusions

Minoxidil, both topical and oral, may be beneficial in monotherapy in patients with alopecia areata. Five percent topical minoxidil is characterized by significantly higher efficacy compared to minoxidil at a lower concentration. There are no sufficient data to recommend minoxidil as a first-line therapeutic option for alopecia areata. Minoxidil can be recommended primarily in combined therapy, while the use of minoxidil in monotherapy should be limited to patients in whom other therapy is contraindicated.

## Figures and Tables

**Table 1:** Summary on characteristics of the selected studies.

Study	Study Design	Concentration (%)/Dose (mg)	Duration of Treatment (Weeks)	Number of Patients (n)	Men (n)	Mean Age (Years)	Type of Alopecia			Responders (n)
							Patchy (n)	Totalis (n)	Universalis (n)	
	Topical Minoxidil									
El Taieb et al. [14]	Randomized controlled trial	5%	12	30	14	26.3	25	2	3	20 (67%)
Ghassemi et al. [15]	Randomized controlled trial	5%	2	18	4	31.7	18	0	0	18 (100%)
El-Ashmawy et al. [16]	Randomized controlled trial	5%	20	20	16	32.2	20	0	0	16 (80%)
Toma et al. [17]	Randomized controlled trial	5%	12	25	NR	NR	25	0	0	23 (92%)
Price et al. [18]	Controlled clinical trial	3%	64	29	16	NR	20	NR	NR	13 (45%)
Khoury et al. [19]	Randomized controlled trial	5%	24	9	5	NR	9	0	0	3 (33%)
Fiedler-Weiss et al. [20]	Controlled clinical trial	5%	60	47	NR	NR	NR	NR	NR	40 (85%)
Price et al. [21]	Controlled clinical trial	3%	48	11	NR	NR	NR	NR	NR	7 (64%)
Price et al. [21]	Controlled clinical trial	5%	48	25	NR	NR	NR	NR	NR	21 (84%)
Fiedler-Weiss et al. [22]	Controlled clinical trial	1%	NR	48	NR	NR	NR	NR	NR	25 (52%)
Ranchoff et al. [23]	Controlled clinical trial	3%	60	25	13	NR	7	10	14	16 (64%)
Maitland et al. [24]	Controlled clinical trial	1%	NR	28	10	37.2	15	6	7	19 (68%)
Frentz et al. [25]	Controlled clinical trial	1%	12	23	13	29	14	2	7	13 (57%)
	Oral Minoxidil									
Fiedler-Weiss et al. [26]	Controlled clinical trial	5 mg	24	34	15	31.1	NR	NR	NR	28 (82%)

NR—not reported.

**Table 2:** Treatment outcomes based on percentage hair regrowth.

Study	Topical Minoxidil	Duration of Treatment [Weeks]	Number of Patients	Number of Patients with Hair Regrowth Equal or Less Than 30% (%)	Number of Patients with Hair Regrowth 30–70% (%)	Number of Patients with Hair Regrowth Equal or More Than 70% (%)
Toma et al. [17]	5%	12	25	6 (24)	6 (24)	13 (52)
Price et al. (2a) [21]	3%	48	11	4 (36)	4 (36)	3 (27)
Ranchoff et al. [23]	3%	60	22	15 (68)	4 (18)	3 (14)

## Data Availability

Not applicable.
